# Potential Effects of Nonadherent on Adherent Human Umbilical Venous Endothelial Cells in Cell Culture

**DOI:** 10.3390/ijms22031493

**Published:** 2021-02-02

**Authors:** Christian Schulz, Anne Krüger-Genge, Andreas Lendlein, Jan-Heiner Küpper, Friedrich Jung

**Affiliations:** 1 Fraunhofer Project Group PZ-Syn of the Fraunhofer Institute for Cell Therapy and Immunology, Branch Bioanalytics and Bioprocesses (IZI-BB), 14476 Potsdam-Golm, Germany, Institute of Biotechnology, Brandenburg University of Technology Cottbus-Senftenberg, 01968 Brandenburg, Germany; Christian.Schulz@izi-bb.fraunhofer.de; 2 Institute of Active Polymers and Berlin-Brandenburg Center for Regenerative Therapies, Helmholtz-Zentrum Geesthacht, 14513 Teltow, Germany; krueger.anne@yahoo.de (A.K.-G.); friedrich.jung@b-tu.de (F.J.); 3 Department of Anesthesia, Pain Management and Perioperative Medicine, Faculty of Medicine, Dalhousie University, Halifax, NS 6299, Canada; 4 Institute of Chemistry, University of Potsdam, Karl-Liebknecht-Straße 24-25, 14469 Potsdam, Germany; 5 Institute of Biotechnology, Molecular Cell Biology, Brandenburg University of Technology, 01968 Senftenberg, Germany; Jan-Heiner.Kuepper@b-tu.de

**Keywords:** human venous endothelial cells, adherent, nonadherent, viability, mediator release

## Abstract

The adherence and shear-resistance of human umbilical venous endothelial cells (HUVEC) on polymers is determined in vitro in order to qualify cardiovascular implant materials. In these tests, variable fractions of HUVEC do not adhere to the material but remain suspended in the culture medium. Nonadherent HUVEC usually stop growing, rapidly lose their viability and can release mediators able to influence the growth and function of the adherent HUVEC. The aim of this study was the investigation of the time dependent behaviour of HUVEC under controlled nonadherent conditions, in order to gain insights into potential influences of these cells on their surrounding environment in particular adherent HUVEC in the context of in vitro biofunctionality assessment of cardiovascular implant materials. Data from adherent or nonadherent HUVEC growing on polystyrene-based cell adhesive tissue culture plates (TCP) or nonadhesive low attachment plates (LAP) allow to calculate the number of mediators released into the culture medium either from adherent or nonadherent cells. Thus, the source of the inflammatory mediators can be identified. For nonadherent HUVEC, a time-dependent aggregation without further proliferation was observed. The rate of apoptotic/dead HUVEC progressively increased over 90% within two days. Concomitant with distinct blebbing and loss of membrane integrity over time, augmented releases of prostacyclin (PGI2, up to 2.91 ± 0.62 fg/cell) and platelet-derived growth factor BB (PDGF-BB, up to 1.46 ± 0.42 fg/cell) were detected. The study revealed that nonadherent, dying HUVEC released mediators, which can influence the surrounding microenvironment and thereby the results of in vitro biofunctionality assessment of cardiovascular implant materials. Neglecting nonadherent HUVEC bears the risk for under- or overestimation of the materials endothelialization potential, which could lead to the loss of relevant candidates or to uncertainty with regard to their suitability for cardiac applications. One approach to minimize the influence from nonadherent endothelial cells could be their removal shortly after observing initial cell adhesion. However, this would require an individual adaptation of the study design, depending on the properties of the biomaterial used.

## 1. Introduction

Coronary artery disease is the major cause of mortality and morbidity in industrialized nations. Beside coronary angioplasty/stenting, bypass surgery is frequently performed [1,2,3]. For bypass surgery, autologous grafts are preferably used. However, 5–30% of patients have no suitable veins/arteries available due to previous operations or diseased vessel walls [4]. Therefore, synthetic grafts from polymeric biomaterials, such as poly(tetrafluoro ethylene) or poly(ethylene terephthalate), were developed as long-term implant. Every cardiovascular prosthetic implant has thrombogenic potential, which bears the risk of thromboembolic complications [5,6]. Nevertheless, polymeric grafts are useful for large arteries providing long-term patency. Small caliber grafts with diameters <6 mm still are a challenge in biomaterial research [7,8]. Platelet adherence and aggregation can lead to thrombus formation in the lumen of low diameter polymeric grafts up to total occlusion or embolization after thrombus detachment [9] when the blood flow is high enough. A strategy, which is explored in order to gain long-term antithrombogenicity of cardiovascular grafts, are biomaterial surfaces, which are covered in vivo by a confluent, functional cell monolayer [10,11,12,13].

Before novel devices are considered for in vivo implantation, they usually undergo a series of in vitro studies to investigate relevant material properties with respect to their functionality and safety in contact with blood (i.e., thrombogenicity, complement activation) [14]. Since an intact endothelial cell monolayer is the ideal antithrombogenic surface for blood contact, the initial biological evaluation of novel implant materials should comprise in vitro biofunctionality assays evaluating endothelial cell adhesion. However, many biomaterial surfaces often allow only slow or incomplete endothelialization, which frequently is not shear-resistant [15,16] and endothelial cells frequently remain in the supernatant medium for a longer period. Thus far, it has not been sufficiently investigated to what extent these cells can influence the results of in vitro biomaterial evaluation studies. For such biofunctionality assays human umbilical venous endothelial cells (HUVEC) are widely used in vitro as primary cell model in biomaterial research [17].

HUVEC cultured under conditions that prevent adherence and spreading stop growing and lose viability [18,19,20]. The loss of matrix contact has been described as an extracellular signal that induces anoikis [20,21,22], a process frequently observed in vivo in the context of angiogenesis and tumour metastasis [23]. It has been described, that in vitro apoptotic HUVEC release TGF-β, which can act on other HUVEC via the caspase-3 signalling pathway and modulate further apoptotic processes [24]. Furthermore, incubation of viable adherent HUVEC with conditioned medium of apoptotic HUVEC caused the progressive proteolytic release of stored TGF-β from the ECM. It is not yet completely clear whether this occurs in the course of TGF-β secretion or through other cytotoxic cellular components, which possibly arise in the course of progressive necrosis of the apoptotic HUVEC.

Concerning these cellular components, endothelial cell based in vitro models for biomaterial evaluation provide only limited capability for clearance of apoptotic cells or cell fragments, comprising the possibility for the accumulation of cell remnants and mediators, which may can stimulate degenerative or even regenerative processes in nearby viable cells. Recently, it was shown, that apoptotic HUVEC release two types of extracellular vesicles upon caspase-3 activation: apoptotic bodies and exosome-like nanovesicles loaded with immunostimulatory RNAs causing inflammation after injection into mice [25]. Further, apoptotic bodies from endothelial cells, packed with miRNA were shown to act as an extracellular signal for surrounding vascular cells (i.e., endothelial cells, smooth muscle cells) inducing CXCL12 expression, which acted chemotactic for progenitor cells in mice reducing atherosclerosis in vivo [26]. 

It is clear from previous studies, that nonadherent HUVEC can have a major impact on the endothelialization of biomaterial surfaces. However, a possible release of signaling molecules from nonadherent dying HUVEC was often neglected in previous biomaterial endothelialization studies, which may have led to misleading conclusions for the in vitro assessment of cardiovascular implant material candidates. Therefore, HUVEC survival rate, aggregate formation and mediator release in anoikis are highly relevant aspects for initial in vitro biomaterial evaluation. The decision for further use of a candidate material may depend on these experiments and an inaccurate assessment of the endothelialization potential could exclude a promising biomaterial from further investigation. Therefore, all influencing factors present in the test system should always be taken into account for a secure evaluation. We addressed this topic in our study by investigating HUVEC behaviour under nonadherent conditions within the initial period of an in vitro biomaterial endothelialization study, concerning time dependent progress of HUVEC proliferation, anoikis, inflammatory activation and secretion of mediators, which may influence adherent HUVEC in the same cell culture.

## 2. Results

### 2.1. Study Design

**Figure 1:**
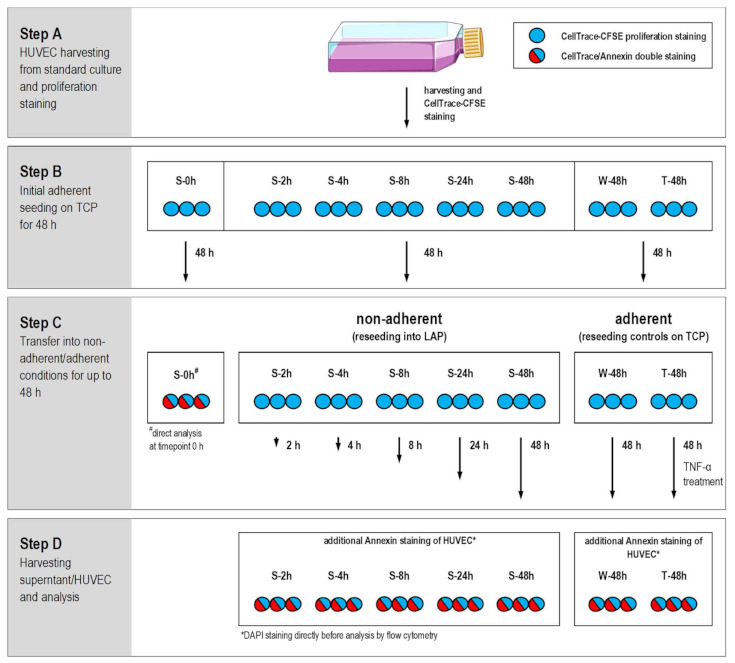
Design of the comparative study cultivating HUVEC in parallel under nonadherent and adherent conditions for up to 48 h is shown for one experimental repeat with three HUVEC seedings (*n* = 3). The study was performed in the six well format with all together six HUVEC seedings for each sample in two independent experiments (*n* = 6 in sum). Samples were S-2h till S-48h for sedimented HUVEC on low attachment plates (LAP) for periods up to 48 h in step C, whereby S-0h was analysed directly after step B. Periods in suspension are indicated by the time code of the sample description (S-2h, S-4h, S-8h, S-24h and S-48h). HUVEC controls W-48h and T-48h were grown on TCP for 48 h during step C, whereby T-48h samples were additionally treated with rhTNF-α to induce HUVEC inflammatory activation and apoptosis (Cell culture flask picture modified from Servier Medical Art; Licence: CC BY 3.0; https://smart.servier.com/).

In this study, the cellular aspect regarding delayed or incomplete endothelialization was considered in the context of the in vitro evaluation of biomaterial surfaces. The focus was placed on the behaviour of nonadherent endothelial cells in the time frame of a rotational medium change in order to gain insights into possible influences of these cells on their surrounding environment under standardized conditions. In the study, polystyrene-based cell culture materials were used, which are established in in vitro cell culture systems. In tissue culture plates (TCP) the surface is optimised for most adherent cells due to its high hydrophilicity and surface charge. Low attachment plates (LAP) are hydrogel coated polystyrene used for suspension cell models and effectively prevent cell adhesion due to their hydrophilic, nonionic and neutrally charged surface properties. Both material surfaces thus represent the extremes in terms of endothelialization and were therefore deliberately used in this study to determine the behaviour of nonadherent HUVEC, without subjecting the cells to serum deprivation, as has been done in other studies to induce anoikis [24]. HUVEC serum starvation would not have comparably simulated the cultivation conditions that would prevail in the in vitro assessment of a candidate material for implants. Therefore, after a detailed understanding of this system, other materials can be investigated. In this study, a controlled comparison of adherent and nonadherent HUVEC was performed with *n* = 6 for each condition in two independent experimental series with three HUVEC seedings each according to the scheme displayed in Figure 1. Detailed methodical information is described in the method section (see Section 4.3)

In step A, HUVEC from standard culture were labelled with CellTrace-CFSE proliferation marker (see Section 4.4) and seeded for step B in six well tissue culture plates (TCP) to prove normal proliferative cell behaviour under standard culture conditions prior to the sedimentation part of the study, starting with step C. After 48 h under adherent conditions HUVEC were harvested by trypsin/EDTA treatment and used for the comparison between adherent and nonadherent culture conditions.

In step C, HUVEC controls (W-48h and T-48h) were reseeded on TCP for 48 h until analysis. W-48h served as growth control for adherent HUVEC under standard cell culture conditions, whereby T-48h samples were additionally treated with recombinant human TNF-α (rhTNF-α) as positive control to induce inflammatory activation and artificial apoptosis induction. Nonadherent HUVEC samples S-2h till S-48h were reseeded into six well low attachment plates (LAP), where they remained in suspension without adhering for up to 48 h. Periods in suspension are indicated by the time code of the sample description (S-2h, S-4h, S-8h, S-24h and S-48h). S-0h samples were used for HUVEC analysis directly before transfer in nonadherent conditions.

In step D, supernatant and HUVEC were harvested for analysis of different parameters, such as cell morphology, proliferation, viability status, cell integrity, and mediator release.

### 2.2. HUVEC Adhere and Proliferate on TCP

In prior to the comparative study performed experiments with HUVEC grown on TCP, automated cell counting showed that HUVEC were 99% adherent after 48 h and only a small percentage of cells remained nonadherent in the supernatant (adherent: 4.8 ± 0.6 · 10^5^ cells/mL; supernatant: 4.9 ± 2.5 · 10^3^ cells/mL). About 86% of HUVEC in the supernatant showed a positive trypan blue staining indicating a high fraction of dead cells (dead: 4.2 ± 2.2 · 10^3^ cells/mL; viable: 0.7 ± 0.5 · 10^3^ cells/mL). In contrast, only four percent of the adherent HUVEC were dead (viable: 4.9 ± 0.6 · 10^5^ cells/mL; dead: 1.8 ± 0.4 · 10^4^ cells/mL), which would remain in culture after medium change.

### 2.3. Decrease of Proliferation Rate and High Fraction of Dead HUVEC at Nonadherent Conditions

**Figure 2:**
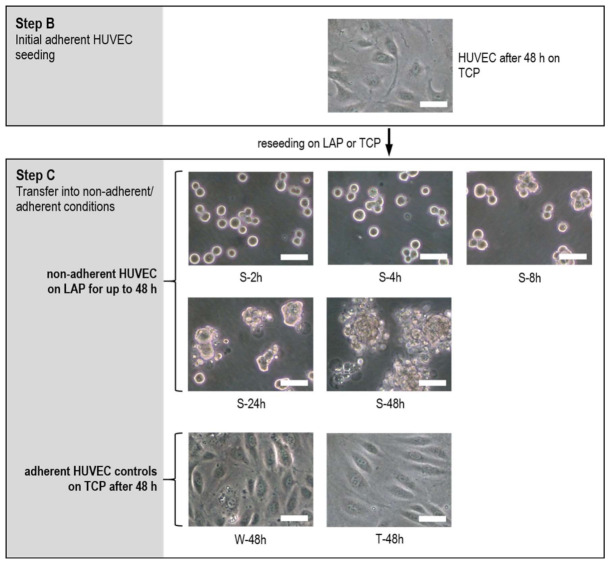
Increasing aggregation and progressing loss of cell integrity of HUVEC under nonadherent conditions on LAP for up to 48 h (S-2h till S-48h) compared to adherent grown controls W-48h and T-48h on TCP. Pictures taken by light microscopy in phase contrast mode at 20-fold primary magnification (scale bar: 50 µm).

Prior to transfer from adherent into nonadherent conditions in step C, HUVEC showed the characteristic morphology of a pre-confluent endothelial cell layer with distinct signs of migration, e.g., marked pseudopodia formation (Figure 2). Under nonadherent conditions, HUVEC tended to form small aggregates within eight hours. Some HUVEC already showed first signs of integrity loss by blebbing and release of small fragments. Over time, HUVEC aggregation and blebbing progressively increased until the end of the study and the number of intact single HUVEC decreased to virtually zero. After the same time period (48 h) adherent HUVEC grown on TCP (W-48h) exhibited cobble-stone pattern of a nearly confluent cell layer. rhTNF-α treated HUVEC (T-48h) differed morphologically by distinct spindle shape and showed a remarkable lower cell density with increased numbers of nucleoli per cell.

**Figure 3:**
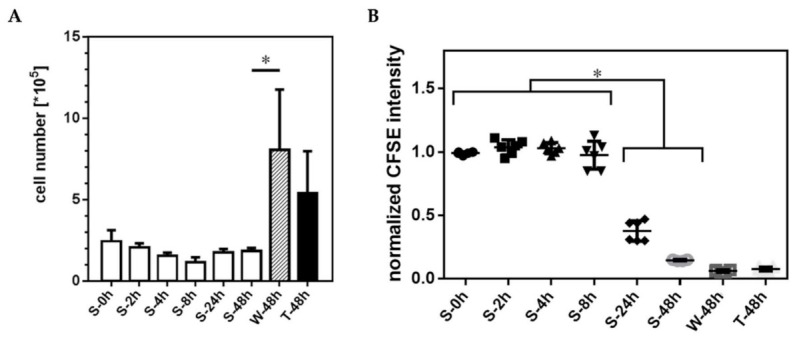
Proliferation of HUVEC under nonadherent conditions. (**A**) In contrast to controls cultured on TCP (W-48h, T-48h), total HUVEC numbers remained unchanged under nonadherent conditions on LAP, while the corresponding signal of the CellTrace-CSFE proliferation marker (**B**) progressively decreased during the cultivation period (Data shown as arithmetic mean ± standard deviation; * *p* < 0.05; *n* = 6 from two independent experimental series with *n* = 3 HUVEC seedings each).

During the initial 48 h cultivation phase of HUVEC on TCP, which was the same for all samples and controls, CellTrace-CFSE proliferation marker intensity decreased over time until transfer of HUVEC into nonadherent conditions. Within this period the marker’s fluorescence intensity decreased from 2089 ± 135 RFU for freshly stained HUVEC to 85 ± 50 RFU after 48 h on TCP. The total cell number of initially seeded 10,500 HUVEC/cm^2^ more than doubled in this period with 25,263 ± 7368 HUVEC/cm^2^ at time point S-0h (S-0h: Start of study with nonadherent HUVEC, Figure 3A). No significant changes in HUVEC numbers occurred during the following 48 h incubation under nonadherent conditions. In contrast, controls on TCP showed a strong increase in HUVEC numbers during this period (85,263 ± 38,947 cells/cm^2^ on TCP (W-48h) and 56,842 ± 27,368 cells/cm^2^ for the rhTNF-α treated HUVEC (T-48h)). During nonadherent conditions CellTrace-CFSE staining intensity remained constant during the first eight hours of sedimentation, but then progressively decreased over time (Figure 3B). After 48 h only negligible difference for the proliferation marker intensity was detectable between HUVEC under nonadherent and adherent conditions.

**Figure 4:**
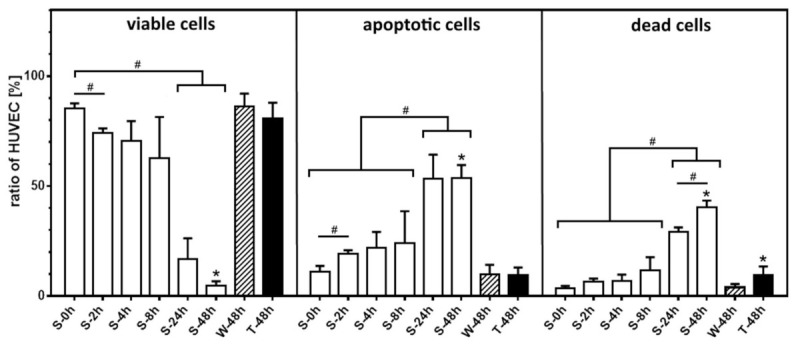
Decrease of HUVEC viability and high fraction of apoptotic/dead cells at nonadherent conditions over time. Time-depended distribution of nonadherent HUVEC viability status (viable = Annexin-V and DAPI negative, apoptotic = Annexin-V positive and DAPI negative, dead = Annexin-V and DAPI positive) compared to TCP grown control (W-48h) and with supplementation of rhTNF-α (T-48h) as positive control for artificial apoptosis induction. Data shown as arithmetic mean ± standard deviation; * *p* compared to growth control on TCP (W-48h); # *p* compared in between samples of nonadherent HUVEC; *n* = 6 from two independent experimental series with *n* = 3 HUVEC seedings each.

**Figure 5:**
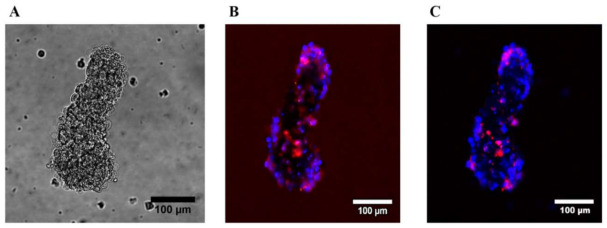
Representation of a multicellular HUVEC aggregate after 24 h (S-24h) under nonadherent conditions. (**A**) Visualized by phase contrast microscopy. (**B**) Same HUVEC aggregate illustrated by cLSM labelled with Annexin-V/Alexa647 for apoptotic (red) and DAPI for dead cells (blue) as overlay of all 60 stacks using maximum projection mode. (**C**) Centre stack of the same HUVEC aggregate illustrated by cLSM.

The viability of HUVEC decreased two hours after transfer into nonadherent culture, when compared to HUVEC grown on TCP (Figure 4). Within 48 h the fraction of nonadherent viable HUVEC decreased to 4.7 ± 2.0%. In the same period, the rate of apoptotic/dead HUVEC increased inversely. In detail, the percentage of apoptotic and dead nonadherent HUVEC increased within 48 h after cell seeding from 10.9 ± 2.8% to 53.4 ± 6.1% and from 3.4 ± 1.2% to 40.3 ± 3.1%. Analysis by confocal laser scanning fluorescence microscopy (cLSM) of nonadherent HUVEC after 24 h in culture showed a minor survival rate of single HUVEC. Within aggregates, most cells showed positive staining for apoptosis and cell death. Most HUVEC were DAPI positive and thus dead, a few cells in the aggregates showed solely a signal for Annexin-V being apoptotic (Figure 5). Most dead HUVEC were found in the center of the aggregate (stack 30 of 60 of the z-stack picture).

### 2.4. Decreasing Cell Membrane Integrity and Accumulative Mediator Secretion of Nonadherent HUVEC

**Figure 6:**
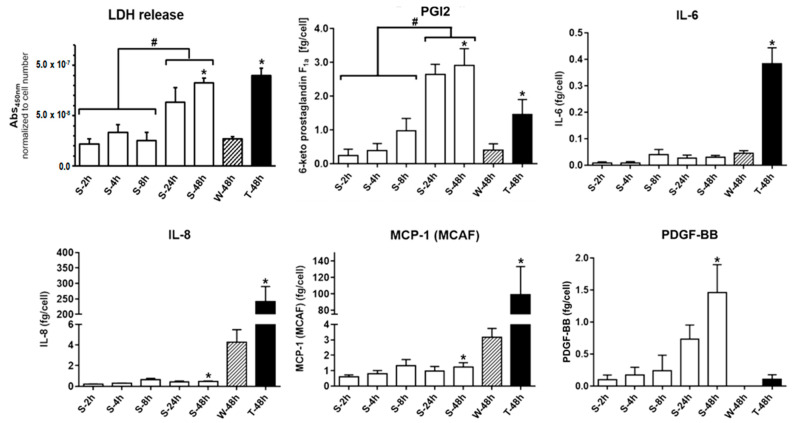
Secretion profile of nonadherent HUVEC. Nonadherent HUVEC release of LDH and various inflammatory mediators, cytokines and growth factors per cell detected in the HUVEC culture medium during cultivation period. Data shown as arithmetic mean ± standard deviation; * *p* compared to growth control on TCP (W-48h); # *p* compared in between nonadherent HUVEC; *n* = 6 from two independent experimental series with *n* = 3 HUVEC seedings each.

Lactate dehydrogenase (LDH) activity was determined in the HUVEC supernatant (Figure 6) in order to investigate, whether nonadherent HUVEC lose membrane integrity over time. With increasing incubation time under nonadherent conditions an increase of LDH activity was detectable, which directly corresponded to the LDH release from HUVEC losing membrane integrity. LDH activity normalized to HUVEC number was for nonadherent HUVEC comparable to HUVEC grown on TCP within the initial eight hours of sedimentation. After 48 h the LDH signal increased significantly up to 3-fold compared to TCP grown HUVEC (S-48h: 8.3 ± 0.5 · 10^−8^ Abs_450nm_/cell (*p* < 0.0001); W-48h: 2.7 ± 0.2 · 10^−8^ Abs_450nm_/cell). Solely rhTNF-α treated adherent HUVEC showed always a higher LDH signal compared to all other samples (T-48h: 9.0 ± 0.7 · 10^−8^ Abs_450nm_/cell).

Vasoactive mediators—prostacyclin (PGI2) and thromboxane A2 (TXA2)—as well as relevant pro- and anti-inflammatory mediators, cytokines, chemokines and growth factors in the supernatant were quantified and when reasonable calculated as average secretion per cell, allowing a comparison between adherent and nonadherent HUVEC (Figure 6). There were no physiologically relevant amounts of thromboxane A2, IL-1ra, IL-12 and VEGF detectable in the supernatant, neither for nonadherent nor for adherent HUVEC at any time point. Secretion of G-CSF and GM-CSF were inducible by rhTNF-α, but there was no difference between detected amounts in the supernatant of HUVEC grown on TCP and LAP. PGI2 accumulated over time in the cell culture medium and was after 48 h under nonadherent conditions more than 7-fold higher compared to HUVEC grown on TCP and doubled compared to rhTNF-α treated HUVEC (S-48h: 2.91 ± 0.62 fg/cell; 8h (TCP): 0.40 ± 0.19 fg/cell; T-48h (TCP + rhTNF-α): 1.46 ± 0.44 fg/cell). This was similar for the accumulation of the growth factor PDGF-BB under nonadherent conditions, while no considerable release could be detected for HUVEC grown on TCP (S-48h: 1.46 ± 0.42 fg/cell; W-48h (TCP): not detectable; T-48h (TCP + rhTNF-α): 0.11 ± 0.07 fg/cell). Minor amounts of the inflammatory mediators IL-6, IL-8 and the chemokine MCP-1 were detectable from nonadherent HUVEC. The amounts of these mediators were similar (IL-6) or increased (IL-8 and MCP-1) for HUVEC grown on TCP.

## 3. Discussion

Blood-material interactions are critical to the clinical success of cardiovascular devices like vascular grafts. Among other complications, thrombosis and clot formation remain to be major challenges in clinical application of vascular grafts [9,27]. One strategy to overcome this problem is the ex vivo or in situ endothelialization of blood contacting implant surfaces. Endothelial cells in a nonactivated state generate or present no or only sparse amounts of coagulation-activating factors [12,28], and were reported to be, not least through the formation of the glycocalyx, the most hemocompatible surface known [29]. Therefore, polymer substrates intended to be used for cardiovascular implants are tested in endothelial cell culture models [17]. Depending on the polymer surface characteristics endothelial cells form a monolayer, or only cell islands, or just colonize as singular endothelial cells [30]. Nonadherent or suspended endothelial cells rapidly lose viability with a half-life of ~10 h [31]. During the apoptotic process, the membrane integrity is slowly reduced until phagocytosis of the detached apoptotic bodies occur. Ultimately, the membrane disintegrates releasing the cellular ingredients in the final stage [32]. Endothelial microparticles are released and phosphatidylserine is exposed on the top of the membrane surface [33]. These microparticles can act as relevant mediators of pro-coagulant processes and inflammation [34] by releasing various factors involved in coagulation such as von Willebrand factor, factor V/Va or tissue factor [35,36,37]. The accumulation of the released mediators in vitro could lead to an incorrect assessment of the endothelialization potential of cardiovascular implant materials in static testing.

The study revealed that HUVEC undergo under nonadherent conditions phenotypic changes. Without the ability to adhere to the biomaterial matrix surface HUVEC progressively formed aggregates within a few hours and showed early evidence for apoptosis. HUVEC viability was significantly decreased already after two hours. Compared to TCP grown HUVEC, nonadherent endothelial cells showed higher numbers of Annexin-V and DAPI positive cells after eight hours, accompanied by distinct blebbing and increased occurrence of cell debris (see Figure 2 and Figure 4). Within 48 h of incubation, which represents the standard cell culture time frame until culture media exchange [17], more than 90% of the HUVEC had entered the apoptotic process, while nearly 40% were already dead. These observations were reinforced by the increase in the LDH activity—used as marker of cell damage and cell death [38]—in the supernatant of nonadherent HUVEC after 24 h in culture and beyond, which suggests a progressive loss of cell membranes functional integrity over time.

The proliferation studies showed a time-dependent decrease of the proliferation marker intensity, which indicated in the first hours of cultivation a proliferation of the nonadherent HUVEC comparable to HUVEC grown on TCP. However, stagnant HUVEC number under nonadhering conditions, progressive loss of membrane integrity and increasing rates of apoptosis and cell death demonstrated the fatal HUVEC development. In our study, about 40% of the HUVEC showed evidence for apoptosis/cell death already after eight hours and nearly 90% after 24 h. In HUVEC cultures grown at nonadherent conditions, apoptosis/cell death events were associated with significant releases of physiologically relevant amounts of the vasodilator PGI2 and the growth factor PDGF-BB. The accumulation of these mediators, but no secretion of, e.g., IL-6 or IL-8 in the supernatant suggests that the stress exerted by the condition of nonadherence did not imply an inflammatory activation. Such an inflammatory response could be demonstrated by treating the HUVEC with rhTNF-α, leading to an upregulation of the secretion of inflammatory mediators such as IL-6, IL-8 or the chemokine CCL2 (MCP-1) and subsequent induction of apoptosis. The baseline values for PGI2 were well in line with earlier in vitro studies with adherent HUVEC [15,39,40]. The increasing accumulation of PGI2 and PDGF-BB over time in culture in case of the nonadherent, dying HUVEC indicated that these mediators were passively released from intracellular storage in the course of progressing membrane integrity loss and degradation. To prove whether it was a passive release, further studies have to be performed regarding the expression profile of nonadherent HUVEC on the transcriptional and translational level.

With respect to in vitro biomaterial evaluation studies, the release of PGI2 by nonadherent HUVEC is expected to influence surrounding viable adherent endothelial cells. PGI2 can induce the release of VEGF [41] followed by an increased proliferation of viable adherent endothelial cells [42]. This effect could be strengthened further by the second mediator released, PDGF-BB. PDGF-BB is known to act as a mitogen and to stimulate endothelial proliferation [43]. Both mediators act in a paracrine manner and support adherent endothelial cells to regenerate the endothelial cell monolayer. In addition, in the case of studies for biomaterial evaluation in co-culture in vitro or angiogenic studies in vivo high local concentrations of unbound PGI2 and PDGF-BB can have considerable influence on the surrounding blood cells as well as on vascular wall cells [44,45,46]. However, further studies are needed to verify these assumptions. This also applies to the question of whether the release of mediators was mainly due to apoptotic HUVEC themselves or possibly induced by apoptotic HUVEC from still vital HUVEC, perhaps localized in cell aggregates, in the cell supernatant. An indication for this could be, that even at the end of the incubation period after 48 h, a small proportion of HUVEC was still vital, so that this cannot be completely excluded. (see Figure 4) Here, the knowledge gained from this study will serve as a basis for defining further scientific questions and conducting corresponding studies. This includes investigations addressing the effects of endothelial cell components from the apoptosis/degradation process (apoptotic bodies alone and/or supernatants with released mediators) on cell viability and proliferation behaviour of an intact endothelial layer in different stages of confluence. Thus, the interplay between potentially proliferation-inducing mediators and possibly proliferation influence in cell remnants have to be investigated in more detail in the future.

The current study clearly revealed that PGI2 and PDGF-BB were mainly released by the nonadherent HUVEC (PGI2: 7-fold higher and PDGF-BB only detected for nonadherent HUVEC) and not by the adherent HUVEC. In contrast, IL-6, IL-8 and MCP-1 were only released by the adherent HUVEC after inflammatory activation by rhTNF-α (IL-6: 7-fold higher, IL-8: 60-fold higher, MCP-1: 30-fold higher) indicating that TCP did not induce an inflammatory response of the HUVEC [47,48]. Studies of this type allow to assess the performance of artificial vascular implants in the process of growing endothelial cells on the implant surface in vitro before the device implantation in vivo.

In context with earlier studies, HUVEC in anoikis associated with the release of mediators such as TGF-β and cell remnants (i.e., apoptotic bodies and exosome-like nanovesicles), have already been shown to elicit different responses in adherent HUVEC in the immediate environment [24,25,26]. These include modulation of broader apoptotic, inflammatory and, through the secretion of PGI2 and PDGF-BB identified in this study, possibly proliferatory and vasoactive mechanisms. Therefore, in an in vitro endothelialization study for evaluating a potential new biomaterial, which is greatly simplified compared to the in vivo situation, influences on surrounding adherent HUVEC are likely. These may vary in nature and strength for each specific biomaterial. A decisive factor here could be the endothelialization potential of the respective biomaterial surface. The more pronounced the initial cell adherence, the fewer endothelial cells enter anoikis and may be a factor influencing already adherent endothelial cells. However, many biomaterials initially show delayed endothelialization [15,16]. Therefore, it seems reasonable to consider nonadherent cells in an in vitro evaluation of a candidate material for cardiovascular application, at least during the period of functional endothelial cell monolayer formation. A possible consequence of neglecting apoptotic HUVEC or secretion of mediators from them in the in vitro evaluation of implant materials is the under- or overestimation of the materials endothelialization potential. If released mediators of apoptotic HUVEC have a pro-apoptotic effect on adherent HUVEC, as occurs, for example, in a concentration-dependent manner through the release of TGF-β alone [24], this can lead to the early rejection of the biomaterial due to the observed low endothelial cell adherence or shear stability. If HUVEC in anoikis have a rather anti-apoptotic and possibly proliferative effect on remaining adherent HUVEC, perhaps due to the comparatively high local mediator concentrations caused by the in vitro conditions, the endothelialization potential of a material may be overestimated. In a later in vivo situation, these mediators would be much less concentrated locally, so that the endothelialization of the implant may be less pronounced.

**Figure 7:**
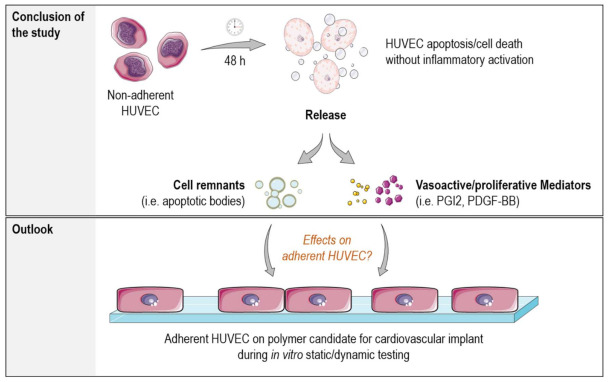
Illustration of the findings from the study. The study revealed that vasoactive and potentially proliferative acting mediators were released by the nonadherent HUVEC over time. There was no evidence for an inflammatory activation of HUVEC solely by cultivation under nonadherent conditions. Further studies will clarify whether mediators released and/or cell remnants from the apoptosis process can influence the surrounding environment and perhaps the endothelialization capacity of potential implant materials during in vitro static and dynamic testing (Picture modified from Servier Medical Art; Licence: CC BY 3.0; https://smart.servier.com/).

Therefore, both static as well as dynamic in vitro endothelialization studies should be considered more critically and in more detail with regard to all cellular factors presented in the experimental setup. (see Figure 7) A possible approach to eliminate endothelialization influencing factors from nonadherent endothelial cells could be their early removal, e.g., by an early medium change after initial cell adhesion. However, generalization of the correct timing is difficult, as different polymeric biomaterials have different surface properties and thus adhesion potential.

## 4. Materials and Methods

### 4.1. Cell Culture

**Figure 8:**
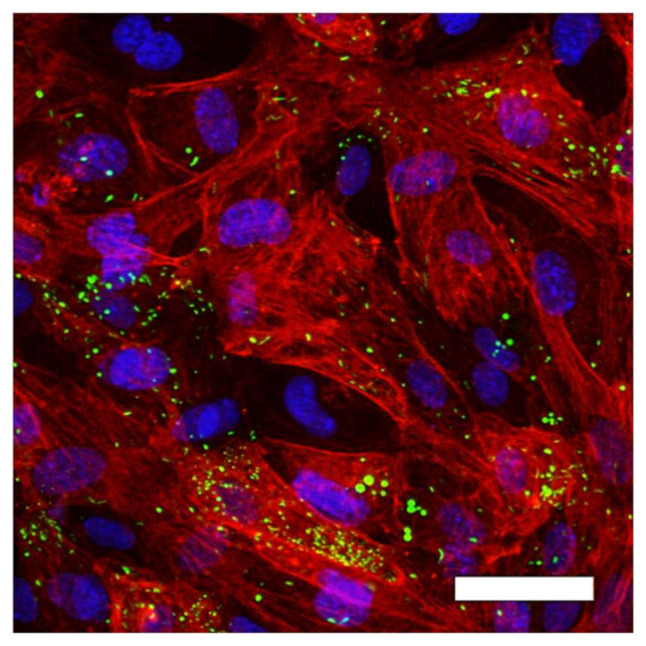
Proof of HUVEC identity by immune fluorescence staining of von Willebrand factor (vWF) expression (green) within adherent HUVEC culture. HUVEC were further stained with DAPI (genomic DNA, blue) and α-SMA (cytoskeleton, red). Picture was taken by cLSM in 20-fold primary magnification; scale bar: 50 µm.

Commercially available primary human umbilical vein endothelial cells (HUVEC, Lonza, Cologne, Germany) were cultivated under static cell culture conditions (37 °C, 5 vol.-% CO_2_) in tissue culture polystyrene flasks (TCP, Techno Plastic Products AG, Trasadingen, Switzerland) with endothelial basal medium EBM-2 supplemented with EGM-2 Single Quots^®^ kit and 2 vol.-% FBS to EGM-2 full medium (Lonza, Cologne, Germany). HUVEC were used for no longer than four passages [49]. All HUVEC and all culture media and culture media supplements were from the same charges. HUVEC identity was proved by immune fluorescent detection of von Willebrand factor expression (Figure 8).

### 4.2. Preliminary Study: HUVEC Adherence on TCP

Prior to the comparative study of HUVEC at nonadherent or adherent conditions, a preliminary study was performed to count adherent and nonadherent as well as viable and dead HUVEC grown on TCP, a material that is often used as control [50]. HUVEC were seeded in 6-well plates with an initial cell density of 10,500 HUVEC/cm^2^ (1 · 10^5^ per well) and incubated under standard cell culture conditions for 48 h. Subsequently, nonadherent HUVEC were harvested by isolation of the supernatant and additionally rinsing of the culture with PBS, whereby the supernatant was pooled with the PBS wash fraction. Remaining adherent HUVEC were harvested quantitatively from the wells by trypsin/EDTA treatment (0.25% *v*/*v* Trypsin and 0.53 mM EDTA in PBS, PAN-Biotech GmbH, Aidenbach, Germany). After centrifugation of the supernatant and the adherent fraction at 220× *g* for 5 min, the cell pellets were resuspended in PBS and the HUVEC were quantified and characterized by trypan blue staining and automated cell counting using a Countess™ II FL Automated Cell Counter (Thermo Fisher Scientific Inc., Waltham, MA, USA).

### 4.3. Studies with Nonadherent HUVEC

Studies with nonadherent HUVEC were realized by transferring freshly CellTrace-CSFE labelled HUVEC (see Section 4.4) with a density of 10.500 cells/cm^2^ from step A in a first incubation period (step B) in six well TCP culture plates (*n* = 6 in two independent experiments, TCP from Techno Plastic Products AG, Trasadingen, Switzerland)), as prove for typical HUVEC adherence and proliferation (see Figure 1). After cultivation under static conditions for 48 h, HUVEC were harvested by trypsin/EDTA procedure described above and subsequently transferred, for the second incubation period (step C), into either six well low attachment plates (LAP, Corning Costar, Merck KGaA, Darmstadt, Germany) for nonadherent HUVEC samples or TCP culture plates for adherent HUVEC controls. As prove for cell proliferation and viability HUVEC grown on TCP served as growth control (W-48h). HUVEC additionally treated with recombinant human TNF-α (rhTNF-α, 200 ng/mL, R&D Systems Inc., Minneapolis, MN, USA), which was added twice 4 and 24 h after seeding, served as positive control for HUVEC inflammatory activation and artificial apoptosis induction. After an incubation period of 2, 4, 8, 24 or 48 h under nonadherent conditions, HUVEC morphology was examined by light microscopy in phase contrast mode (Axiovert 40 C, Zeiss, Oberkochen, Germany). Supernatants (300 µL) from nonadherent and adherent samples were isolated, centrifuged (300× *g* for 5 min) to eliminate remaining cells or debris and stored immediately at −20 °C until secretion profile analysis. Remaining cells/supernatant from nonadherent HUVEC were harvested, quantified and analyzed for proliferation as well as apoptosis/cell death by flow cytometry, according to the protocols described below.

### 4.4. Proliferation and Apoptosis/Cell Death Analyzed by Flow Cytometry and Fluorescence Microscopy

Prior to cultivation for the comparative study, HUVEC were labelled in step A (see Figure 1) with CellTrace-CFSE proliferation marker (CellTrace-CFSE Cell Proliferation Kit, Thermo Fisher Scientific Inc., Waltham, MA, USA) according to the manufacturer’s instructions. Briefly, lyophilized CellTrace-CFSE was reconstituted in 18 µL DMSO and diluted to a 2.5 µM working concentration in PBS (Biochrom AG, Berlin, Germany). HUVEC in a final concentration of 1 · 10^6^ cells/mL were stained for 30 min under slightly shaking in the dark at 37 °C. Subsequently, HUVEC were washed in a four-fold excess of EGM-2 and further used for cell culture as described in the study design (see Section 2.1). After cultivation period of step C, CellTrace-CFSE labelled HUVEC were harvested and the remaining proliferation marker intensity within the cells was analyzed by flow cytometry as described below.

HUVEC apoptosis was detected using an Annexin-V/Alexa647 apoptosis detection assay (Molecular Probes Inc., Eugene, OR, USA). After cultivation period of step C, HUVEC were harvested, washed with cold PBS and 3 · 10^5^ cells were pelleted by centrifugation at 300× *g* for 5 min. Cells were resuspended in Annexin binding buffer and the cell density adjusted to 1 · 10^6^ cells/mL. 15 µL Annexin-V/Alexa647 conjugate were added to the HUVEC suspension, followed by 15 min incubation at ambient temperature in the dark. Subsequently, samples were stocked up to 500 µL with Annexin binding buffer and examined by flow cytometry. For detection of dead HUVEC 6-diamidino-2-phenylindole (DAPI, Carl Roth GmbH, Karlsruhe, Germany) was added in a final concentration of 1 µM prior to flow cytometric measurements. Flow cytometric analysis of HUVEC was performed with a MACSQuant^®^ Analyzer 10 and MACS Quantify 2.6 software (Miltenyi Biotec GmbH, Bergisch Gladbach, Germany). FlowJo software (version: 10.2, FlowJo LLC, Ashland, OR, USA) was used for further data analysis. Data acquisition was performed without exclusion of small particles (i.e., cell debris) to gather information about HUVEC microparticle release, such as apoptotic bodies, during progress of the experiment as well as to detect the appearance of cell aggregates. Calibration beads in the scale between 6 to 15 µm from the Flow Cytometry Size Calibration Kit (Invitrogen AG, Carlsbad, CA, USA) were used as basis for further analysis of HUVEC by flow cytometry concerning apoptosis/cell death as well as proliferation determination by CellTrace-CFSE labelling, to identify the lower size limit of single HUVEC compared to cell debris. Single cell size of viable nonadherent HUVEC was concluded to range between 14 and 19 µm from light microscopy pictures (*n* = 109) and confirmed by published data [51]. Fluorescence microscopic pictures of Annexin-V/Alexa647 and DAPI stained HUVEC were taken by using a confocal laser scanning microscope (cLSM) with a 20-fold primary magnification (LSM 510 META, Zeiss, Oberkochen, Germany).

### 4.5. Membrane Integrity and Secretion Profile of HUVEC

Structural integrity of HUVEC membranes during incubation under nonadherent conditions was examined by lactate dehydrogenase (LDH) activity in the extracellular fluid by using the Cytotoxicity Detection Kit LDH (Roche, Grenzach, Germany) according to the manufacturer’s instructions. Secretion of the antagonistically acting vasoactive mediators prostacyclin (PGI2) and thromboxane A2 (TXA2) was quantified from the supernatant of nonadherent and adherent HUVEC by use of a competitive inhibition enzyme immunoassay for the stable hydrolysis product of PGI2 (6-keto-prostaglandin F1α EIA Kit from Cayman Chemical Company, Ann Arbor, MI, USA) and TXA2 (thromboxane A2 ELISA Kit for Thromboxane A2, Cloud-Clone Corporation, Houston, TX, USA). Similarly, the concentration of prominent pro- and anti-inflammatory mediators (IL-1ra, IL-6, IL-8), cytokines (Il-12, GM-CSF, IFN-γ), chemokines (G-CSF, MCP-1) and growth factors (PDGF-BB, VEGF) were quantified from the supernatant by magnetic bead-based Bioplex™ Cytokine Assay (Bio-Rad Laboratories Inc., Munich, Germany), whereby EGM-2 served always as background control.

### 4.6. Statistical Analysis

For all samples, arithmetic means and standard deviations are given. Data were collected in two experimental series each with three independent HUVEC seedings in separated wells (*n* = 6 in sum). Statistical significance between HUVEC samples in nonadherent conditions was calculated with a one-way ANOVA for repeated measures subsequently followed by Tukey multiple comparison test using Prism 8 (GraphPad software, https://www.graphpad.com/scientific-software/prism/). Further, statistical significance was calculated between S-48h and W-48h as well as W-48h and T-48h respectively with a two-tailed paired t-test. Statistical significance was assumed for *p* < 0.05 for all statistical analyses.

## 5. Conclusions

HUVEC grown under nonadherent conditions started to aggregate and to show evidence for the initiation of programmed cell death already after 2 h in suspension. More than 90% of HUVEC were apoptotic/dead after two days (only 4.7 ± 2.0% of the HUVEC were still viable) and no further proliferation occurred. Combined with progressive blebbing and loss of cell membrane integrity, signaling mediators like PGI2 and PDGF-BB were increasingly released from intracellular storage. However, there was no evidence for an inflammatory activation of HUVEC solely by anoikis induced by cultivation under nonadherent conditions.

These results show that studies for assessing the endothelialization of implant materials in vitro under static conditions, and maybe dynamic conditions as well, should always take into account the fraction of nonadherent HUVEC still suspended in the cell culture medium. HUVEC remaining in suspension can significantly influence the morphology and function of already adherent HUVEC and thus influence the assessment of the endothelialization capacity of implant materials, possibly distorting the assessment of the results.

## Data Availability

The data that support the findings of this study are available from the corresponding author upon reasonable request.

## Abbreviations

HUVEC	Human umbilical vein endothelial cells
TCP	Tissue culture plates
LAP	Low attachment plates
RFU	Relative fluorescence units
FBS	Fetale bovine serum
vWF	von Willebrand factor
rhTNF-α	recombinant human TNF-α
DAPI	6-diamidino-2-phenylindole
LDH	Lactate dehydrogenase
PGI2	Prostacyclin
TXA2	Thromboxane A2
IL-1ra	Interleukin 1 receptor antagonist
IL-6	Interleukin 6
IL-8	Interleukin 8
IL-12	Interleukin 12
G-CSF	Granulocyte-colony stimulating factor
GM-CSF	Granulocyte-monocyte-colony stimulating factor
IFN-γ	Interferone-γ
MCP-1 (CCL-2)	Monocyte chemotactic protein-1 (CC-chemokine-ligand-2)
PDGF-BB	Platelet-derived growth factor BB
VEGF	Vascular endothelial growth factor
